# Striving for improved visibility and increased citation through coverage by PubMed Central (PMC)

**DOI:** 10.12669/pjms.301.4878

**Published:** 2014

**Authors:** Shaukat Ali Jawaid

During the Year 2013 Pakistan Journal of Medical Sciences achieved yet another landmark when it managed to be covered by PubMed Central with the result that now all the manuscripts published in the journal effective January 2013 are now accessible through this important global database i.e. PubMed Central. (http://www.ncbi.nlm.nih.gov/pmc/) It will ensure that the contributions by the authors have increased visibility and readership thereby improving the chances of citation as well. We had been planning for this for the last three years but financial constraints had been the major hindrance. However, now we have made arrangements with one of the commercial concerns overseas who prepares the XML files for submission to PubMed Central which is a pre requisite. We believe this investment is worth and will further improve the standard of the journal and its ranking in biomedical journals. In order to facilitate the authors in early publication of their accepted manuscripts reducing the waiting time, we also increased the frequency of publication from quarterly to bimonthly during 2013 while efforts are also being made to accelerate the Peer Review process and reduce the time but sometimes it is beyond our control since the Reviewers are all busy people and they perform these academic duties in honorary capacity, hence one cannot force them to convey their comments immediately, though many Reviewers are quite efficient and try to complete the review process meeting the deadlines.

**Screening for plagiarism**

Earlier after an agreement with CrossRef we had started giving Digital Object Identifier (DOI) numbers to all the manuscripts. In order to protect the authors and ourselves from publication of plagiarized material, we also entered into an agreement with CrossCheck to use their software iThenticate for screening of manuscripts for plagiarism. This software though a bit expensive but has the advantage over other tools used for screening for plagiarism because this not only covers what is freely available on the internet but it also has access to journal publications that are not accessible free online.^1^ During the last six months, after triage, only one hundred fifty manuscripts which are about 20% of all the submissions were accepted for further processing. Twelve were rejected because during screening similarity index score was more than 20% and in some cases it was as high as 40-60%. While screening for plagiarism, we remove the title as well as references from the manuscript. Three manuscripts which had similarity index score between 20-25% were returned to the authors requesting them to revise them and then resubmit by making necessary changes so that the similarity index score is less than 20%. While looking at the similarity index score one has to be careful as most often the plagiarized parts are in Methods section of the manuscript followed by Discussion. In some cases since the methodology is mostly the same in experimental studies, one has to be a bit lenient and accommodative but extensive plagiarism in discussion is not at all tolerated. 

***Impact Factor:*** We have always believed that Impact Factor is Not and should Not be considered as the only criteria to evaluate the quality and standard of a Journal but it has its own importance and gets weightage from various academic institutions and organizations.^2^ Impact Factor has its own merits, advantages and disadvantages. At times the Editors are in a dilemma while accepting manuscripts for publication because in order to increase their Impact Factor, they are more interested to accept, process and ensure early publication of those manuscripts which have higher chances of citation. Case Reports get the least priority. On the other hand, impatience by the authors who wish to see their manuscripts in print early, the few quality journals are under too much pressure to publish more manuscripts once they are approved after going through the peer review. Increased submissions leads to increased number of manuscripts which eventually get accepted after peer review and thus publication but it then adversely affects the Journal’s Impact Factor. Despite the fact that citations from Pakistan Journal of Medical Sciences have progressively increase every year since 2009 but its Impact Factor got reduced since we published more manuscripts. Table-I. 

**Table-I:** Number of yearly Citations from Pak J Med Sci and its Impact Factor^3^

*Year*	*No. of Citations*	*Impact Factor*
2009	198	0.203
2010	275	0.166
2011	283	0.161
2012	349	0.101

Since we follow author friendly policy, we do not wish to deprive the authors from publishing their research work just for the sake of Impact Factor but we are hopeful that in the days to come as more and more quality manuscripts will get published, it will also ensure greater citations which will improve this Impact Factor as well in the days to come. However, great care is taken to ensure that only good quality manuscripts are accepted for publication which results in increased rejection rate which is not at all liked for the authors whose manuscripts are not accepted for further processing and publication. But one has to work within the constraints of financial as well as human resources and ensure timely publication through effective and efficient coordination with the authors and reviewers which is not an easy task.

Some scientists have suggested numerous other alternate metrics to judge and evaluate the quality and standard of a journal like H-Index and M-Index but they have their own drawbacks. ^1^ More recently some scientists have hypothesized that “using journal rank as an assessment tool is bad scientific practice”. They have suggested a library-based scholarly communication system. They believe that “this new system which will use modern information technology will vastly improve the filter, sort and discovery functions of the current journal system”. ^4^ They have further pointed out that it is not at all surprising to find out that even higher ranking journals are also more likely to publish fraudulent work than lower ranking journals.^5^ However, we believe that instead of becoming a victim of this vicious circle, editors in the developing resource constraint countries should try to improve the quality and standard of their journals making best use of the available financial and human resources. Consistency and hard work by the journal’s editorial team will pay rich dividends and ensure gradual improvement in journal ranking.

## References

[1] Roosendaal FR, Reitsma Ph (2013). Copy and paste. J Thromb Haemost.

[2] Jawaid SA (2014). Despite misuse and abuse, journal Impact Factor will retain its Impact and won’t fade away soon. J Postgrad Med Inst.

[3] (2013). Journal Citation Report. Thompson/ISI Web of Sciences.

[4] Brembs BN, Btton K, Munafo M (2013). Deep Impat: unintended consequences of journal rank. Frontiers in human Neuroscience.

[5] Fang FC, Steen RG, Casadevall A (2012). Misconduct accounts for the majority of retracted scientific publications. Proc. Natl Acad Sci USA.

